# *PCDH7* as the key gene related to the co-occurrence of sarcopenia and osteoporosis

**DOI:** 10.3389/fgene.2023.1163162

**Published:** 2023-07-05

**Authors:** Mingchong Liu, Yongheng Wang, Wentao Shi, Chensong Yang, Qidong Wang, Jingyao Chen, Jun Li, Bingdi Chen, Guixin Sun

**Affiliations:** ^1^Department of Traumatic Surgery, Shanghai East Hospital, School of Medicine, Tongji University, Shanghai, China; ^2^ Shanghai Tenth People’s Hospital, School of Medicine, Tongji University, Shanghai, China; ^3^ Institute for Regenerative Medicine, Shanghai East Hospital, The Institute for Biomedical Engineering and Nano Science, Tongji University School of Medicine, Shanghai, China

**Keywords:** sarcopenia, osteoporosis, crosstalk genes, machine learning, bioinformatic analysis

## Abstract

Sarcopenia and osteoporosis, two degenerative diseases in older patients, have become severe health problems in aging societies. Muscles and bones, the most important components of the motor system, are derived from mesodermal and ectodermal mesenchymal stem cells. The adjacent anatomical relationship between them provides the basic conditions for mechanical and chemical signals, which may contribute to the co-occurrence of sarcopenia and osteoporosis. Identifying the potential common crosstalk genes between them may provide new insights for preventing and treating their development. In this study, DEG analysis, WGCNA, and machine learning algorithms were used to identify the key crosstalk genes of sarcopenia and osteoporosis; this was then validated using independent datasets and clinical samples. Finally, four crosstalk genes (*ARHGEF10*, *PCDH7*, *CST6*, and *ROBO3*) were identified, and mRNA expression and protein levels of *PCDH7* in clinical samples from patients with sarcopenia, with osteoporosis, and with both sarcopenia and osteoporosis were found to be significantly higher than those from patients without sarcopenia or osteoporosis. *PCDH7* seems to be a key gene related to the development of both sarcopenia and osteoporosis.

## 1 Introduction

In recent years, sarcopenia and osteoporosis, two degenerative diseases in the motor system of older adults, have attracted increasing attention from researchers due to increasingly aging societies (Hu et al., 2022; Khosla et al., 2022). For instance, the proportion of the aged population in South Korea was 16.6% in 2021, and this may reach 25.5% after 10 years and 40.1% after 30 years (Kwon et al., 2022). Across the world, the number of individuals aged more than 65 years outnumbered the number of children in 2018, and it is predicted that this number may double in the next 30 years (Mastnak et al., 2022).

Sarcopenia is an aging syndrome characterized by decreased skeletal muscle mass, function, and strength (Kim et al., 2022). The first practical clinical definition and consensus diagnostic criteria for it were developed in 2010 by the European Working Group on Sarcopenia in Older People (EWGSOP), and the consensus was updated in 2019 (EWGSOP2) (Cruz-Jentoft et al., 2010; Cruz-Jentoft et al., 2019). EWGSOP2 developed and emphasized low muscle strength as a key feature of sarcopenia, muscle content as a diagnostic basis, and physical function as an indicator of sarcopenia severity (Spexoto et al., 2022). An observational study conducted in Spain reported that the prevalence of sarcopenia in older people was 28.9% in men and 26.2% in women (Guillamon-Escudero et al., 2022). Sarcopenia may lack specific clinical features; patients with it may present with weakness, difficulty walking, slow gait, and thin and weak limbs (Aoki et al., 2022). Sarcopenia may increase the risk of falls and fractures (de Almeida Marques Bernabe et al., 2022), decrease the quality of daily living activities, and relate to many comorbidities such as cardiac disease (Loh et al., 2022), diabetes (Jun et al., 2022), and cognitive impairment (Noda et al., 2022)—although these symptoms can be easy to ignore (Malmstrom et al., 2016; Mijnarends et al., 2018).

Osteoporosis is a disease characterized by low bone mass and deterioration of the microstructure of bone tissue (Collins et al., 2017). Its diagnosis is mainly dependent on the evaluation of bone mineral density (BMD); patients with a BMD of more than 2.5 standard deviations lower than the average for young adults may be diagnosed with osteoporosis (Kanis et al., 2019). According to a European Union (EU) survey, there may be 22 million women and 5.5 million men in the EU with osteoporosis (Hernlund et al., 2013). Fragility fractures caused by osteoporosis have become one of the most severe and deadly health problems in the world, and the prevention of osteoporotic fractures has become the most significant aspect of managing the condition in patients (Zhang H. et al., 2022). The commonest site of fragility fractures is the spine, and the deadliest is the hip. Patients with fragility hip fractures had an annual mortality rate of more than 20%, even after surgery (Liu M. et al., 2022).

Muscle and bone are the most important components of the motor system, and patients with both muscle and bone disorders may face worse health and a higher risk of falls and fractures (Cevei et al., 2020). Osteosarcopenia was defined for the first time for patients with both sarcopenia and osteoporosis (Polito et al., 2022). A study conducted in China showed that the prevalence of sarcopenia and osteoporosis in men and women over 65 years old was 10.4% and 15.1%, respectively (Wang et al., 2015). Co-occurrence of sarcopenia and osteoporosis may affect patients additively and synergistically (Clynes et al., 2021; Teng et al., 2021) and contribute to a higher risk of falls and dysfunction as well as poorer outcomes after fractures than those with just one of these diseases (Reiss et al., 2019; Okayama et al., 2022).

Muscle and bone are derived from mesodermal and ectodermal mesenchymal stem cells, with the adjacent anatomical relationship between muscle and bone providing the basic conditions for mechanical and chemical signals (Lam and Qin, 2008). The interaction between bone and muscle is mainly achieved through mechanical stimulation and their secretion of bioactive factors (Tagliaferri et al., 2015). In addition, endocrine, disease, genetics, exercise, and other factors also jointly affect bone and muscle phenotypes (Kawao and Kaji, 2015). In the occurrence and development of osteosarcopenia, the communication of mechanical and chemical signals between muscle and bone may be the pathogenesis of osteosarcopenia, resulting in dysfunction of the skeletal muscle system (Shen et al., 2015). Osteoporosis and sarcopenia have common risk factors, and the reasons behind these factors obscure the pathogenesis of osteosarcopenia (Huang et al., 2014). Therefore, it is significant to identify the correlation and co-pathway between sarcopenia and osteoporosis, and to then explore potential treatment that may improve life quality and decrease the risk of adverse events for patients with it.

**FIGURE 1:**
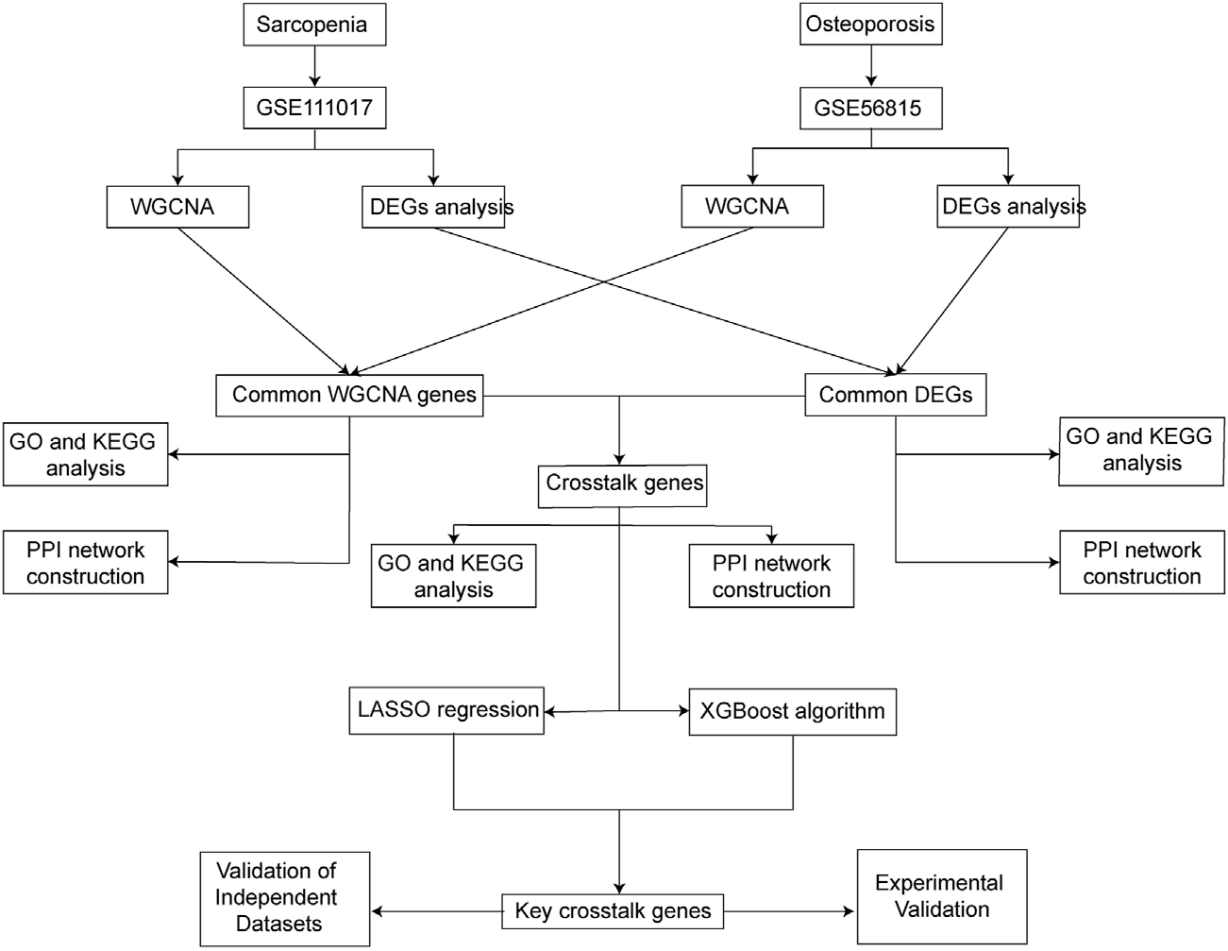
Study’s flow chart.

With the development of microarray technology and high throughput sequencing, more and more data for comparing differentially expressed genes between individuals with different diseases state have become available (Hephzibah Cathryn et al., 2022). Bioinformatic analysis and machine learning algorithms based on these data may provide us with more information about relationships, maps, and expressions of genes and proteins (Cheng et al., 2021). However, few studies have used bioinformatic analysis and machine learning algorithms to explore the common genes that contribute to the co-occurrence of sarcopenia and osteoporosis. In this study, differentially expressed gene (DEG) analysis, weighted gene co-expression network analysis (WGCNA), and two kinds of machine learning algorithms were used to identify the common key crosstalk genes between sarcopenia and osteoporosis. Moreover, experimental validations of expression and protein levels were performed with real clinical samples. The study’s flow chart is in Figure 1.

## 2 Methods

### 2.1 Data downloading and preparation

All datasets included in our study were downloaded and collected from the Gene Expression Omnibus (GEO) database (http://www.ncbi.nlm.nih.gov/geo/). GEO is one of the largest public databases to include microarray data and high-throughput gene expression data submitted by research institutions around the world (Patra et al., 2020). To collect the expression data of sarcopenia and osteoporosis, the keywords “osteoporosis” and “sarcopenia” were searched, and then the datasets “GSE111017,” “GSE56815,” “GSE167186,” and “GSE35956” were included in our study. GSE167186 and GSE35956 as the independent datasets were used to validate the key crosstalk genes that we identified.

### 2.2 DEG analysis

Sangerbox, an online tool based on R, was used to perform the DEG analysis of sarcopenia and osteoporosis datasets (Weitao Shen et al., 2022). GSE111017 is a profile of high-throughput gene expression, and the DEG analysis for GSE111017 was performed by using the DESeq2 package (version 1.32.0), while the limma package was used to conduct the DEG analysis for GSE56815. In the sarcopenia dataset, genes with *p* < 0.05 and fold change >1.2 were defined as DEGs; for osteoporosis, genes with *p* < 0.05 and fold change >1.1 were identified as DEGs. Visualized modules based on the ggplot2 (version 3.3.2) and pheatmap packages (version 1.0.12) in Sangerbox were used to visualize the DEGs.

### 2.3 WGCNA

WGCNA as a novel developed bioinformatic approach aims to find co-expressed gene modules and explore the association between gene networks and phenotypes of interest, as well as a network's core genes (Langfelder and Horvath, 2008). Age and gender are significant factors in the development of sarcopenia and osteoporosis, and the clinical characteristics in datasets GSE111017 and GSE56815 are quite different. Therefore, in our study, WGCNA was performed using the R packages “WGCNA” to identify the sarcopenia and osteoporosis modules and reduce the bias caused by different platforms and databases. First, the outlier sample was identified and eliminated using the Hclust function. Next, soft-thresholding power ranging from 1 to 20 was analyzed and calculated based on the criterion R^2^ > 0.85. Based on the soft-thresholding power calculated previously, co-expression genes were clustered and modulized and the minimum number of module genes was set at 30. The modules with a correlation of more than 80% in sarcopenia and more than 70% in osteoporosis were merged for the second time. Finally, the module eigengene and the correlation between the module eigengenes and clinical data were visualized, and all the module genes with significant correlations with sarcopenia and osteoporosis were identified and exported for further analysis.

### 2.4 Identification of crosstalk genes

After separately identifying DEGs and WGCNA genes of sarcopenia and osteoporosis, these were imported into R software to obtain the intersection. The intersection of the DEGs of sarcopenia and osteoporosis was identified as the common DEGs, and the common WGCNA genes were defined as the intersection of WGCNA genes of sarcopenia and osteoporosis. Finally, the intersection of common DEGs and common WGCNA were collected as the crosstalk genes of sarcopenia and osteoporosis.

### 2.5 Functional enrichment analysis and visualization

Functional enrichment analysis was performed to explore the biological functions and pathways involved in the genes we identified. Biological process (BP), cellular component (CC), and molecular function (MF) from Gene Ontology (GO) were used to describe gene information, functions, and interaction, and the pathway results were obtained from the Kyoto Encyclopedia of Genes and Genomes (KEGG) databases (Kanehisa et al., 2016; Pomaznoy et al., 2018). R package “enrichplot” was used to perform the GO and KEGG analysis and the results of the enrichment analysis were imported to Cytoscape (version 3.9.1) for visualization.

### 2.6 PPI network construction

The protein–protein interaction (PPI) network may reveal the protein interactive relationships. The common DEGs, common WGCNA genes, and crosstalk genes were uploaded to STRING (https://string-db.org/) to construct PPI networks with a confidence of more than 0.7 (Szklarczyk et al., 2019). CytoNSA as a Cytoscape plugin was used to calculate the core protein genes of the PPI network. The size of the nodes in the PPI network was proportional to the significance of genes calculated by CytoNSA.

### 2.7 Construction of machine learning modules and identification of key crosstalk genes

To better screen the key crosstalk genes of sarcopenia and osteoporosis, the XGBoost algorithm and least absolute shrinkage and selection operator (LASSO) regression were conducted based on R packages “xgboost” and “glmnet.” In the XGBoost analysis, the importance of each gene was viewed and collected. The coefficient of punishment (λ) was identified according to the AUC of the modules with different λ, and then the predictive value of genes was exported from the modules with the most suitable λ. The intersection of genes screened by XGBoost and LASSO regression was identified as the key crosstalk genes.

### 2.8 Validation of key crosstalk genes

The expression of key crosstalk genes from GSE167186 and GSE35956 datasets were collected to verify the key crosstalk genes. The *t*-test was used to compare the expression between groups, and all *p*-values < 0.05 were considered statistically significant.

### 2.9 Sample collecting and grouping

All participants were recruited from patients awaiting hip arthroplasty due to hip fractures who were admitted to the Department of Traumatology, Shanghai East Hospital, Tongji University, School of Medicine, Shanghai, China. The study was approved by the Institutional Ethics Committee of East Hospital ([2022] Research Review (No. 278), 7 September 2022) and was performed in accordance with the Declaration of Helsinki. The mean age of patients enrolled in this study was 65 years. Finally, 12 patients who underwent hip arthroplasty were included in our study and divided into four groups according to whether they had sarcopenia and osteoporosis. The diagnosis of sarcopenia and osteoporosis followed the guidelines mentioned previously (Cruz-Jentoft et al., 2019; Zhang H. et al., 2022). Skeletal muscle tissue and bone tissue samples—5 mm × 5 mm × 5 mm each—were collected from the vastus lateralis muscle and femoral head during surgery; due to the nature of joint replacement, this had no impact on the patients. After sample collection, visible fat and blood were removed, and the samples were put into liquid nitrogen and stored at ≤−80°C until analysis.

### 2.10 Total RNA isolation and real-time quantitative PCR analysis

RNA isolation was conducted following the manufacturer’s instructions for RNAeasy™ Animal RNA Isolation Kit with Spin Column (Beyotime, China). cDNA was synthesized following the manufacturer’s instructions for PrimeScript II 1st Strand cDNA Synthesis Kit (Takara, Japan). RNA quantity and integrity were measured with a NanoDrop 1000 (Thermo Scientific). The reaction system for Real-time Quantitative PCR followed the manufacturer’s instructions for SYBR^®^ Premix Ex Taq™ (Takara, Japan). Data were normalized to GAPDH mRNA and analyzed according to the 2^−ΔΔCT^ method. The gene primers are: forward primer TTG​TGG​GAG​CAG​GAG​ACA​AC and reverse primer ACT​CTA​CGA​AAT​GGC​TGT​TTG​C for *PCDH7*; forward primer GAA​AGC​CTG​CCG​GTG​ACT​AA and reverse primer GCC​CAA​TAC​GAC​CAA​ATC​AGA​G for *GAPDH*.

### 2.11 Western blot analysis

Proteins were extracted from tissue homogenate, and the protein concentration was quantified using a BCA kit (Epizyme, China). Total proteins of skeletal muscle and bone were subjected to 12% SDS-PAGE electrophoresis and were then wet migrated to the PVDF membrane. The membrane was sealed with 5% skimmed milk at 37°C for 2 h and then incubated with primary antibody (1:1000; HPA011866, Sigma-Aldrich) at 4°C overnight. The membrane was then washed with TBST three times, 15 min each time, and then cultivated with secondary antibody Goat anti-rabbit IgG with 1:2500 dilution at 25°C for 1 h. Finally, the signal was detected on the Azure imaging biosystem C300 using ECL Kit (Kangweishiji Biotechnology).

### 2.12 Immune infiltration

Immune infiltration analysis was performed to identify the roles of the immune system in the bone and muscle system. The online platform CIBERSORTx (https://cibersortx.stanford.edu/) was used for immune infiltration analysis and then the immune infiltration matrix generated from CIBERSORTx was imported into R software for visualization using the R package “ggplot2.” The differences between the two groups were compared using the Wilcoxon test and the correlation between the 22 infiltrating immune cells and key genes was then visualized.

### 2.13 Statistical analysis

Continuous variables were presented as mean ± standard deviation and were evaluated by independent Student’s t-test for normally distributed variables and by Wilcoxon rank-sum test for non-normally distributed data. All *p*-values < 0.05 were considered statistically significant, and statistical analyses were performed using GraphPad Prism version 8.0.1 (GraphPad Software San Diego, United States) and R software version 4.1.1 (R Foundation for Statistical Computing, Vienna, Austria).

## 3 Results

### 3.1 General information of datasets

In this study, GSE111017 and GSE167186 were used as the datasets of sarcopenia, and GSE56815 and GSE35956 as the datasets of osteoporosis. GSE111017 was a dataset containing three sub-datasets (GSE111006, GSE111010, and GSE111016) of data from a multi-center study in Hertfordshire, Jamaica, and Singapore. All the sub-datasets in GSE111017 were expression profiled by high-throughput sequencing using the same platforms, and the batch effect between sub-datasets was evaluated. From the evaluation results of the batch effect, there was no significant difference between the datasets (Supplementary Figure S1). GSE167186 contains the data of transcriptome profiling on muscle samples from 72 subjects with or without sarcopenia at different ages. GSE56815 and GSE35956 are the datasets with samples from patients with or without osteoporosis.

### 3.2 Identification of common DEGs of sarcopenia and osteoporosis

**FIGURE 2:**
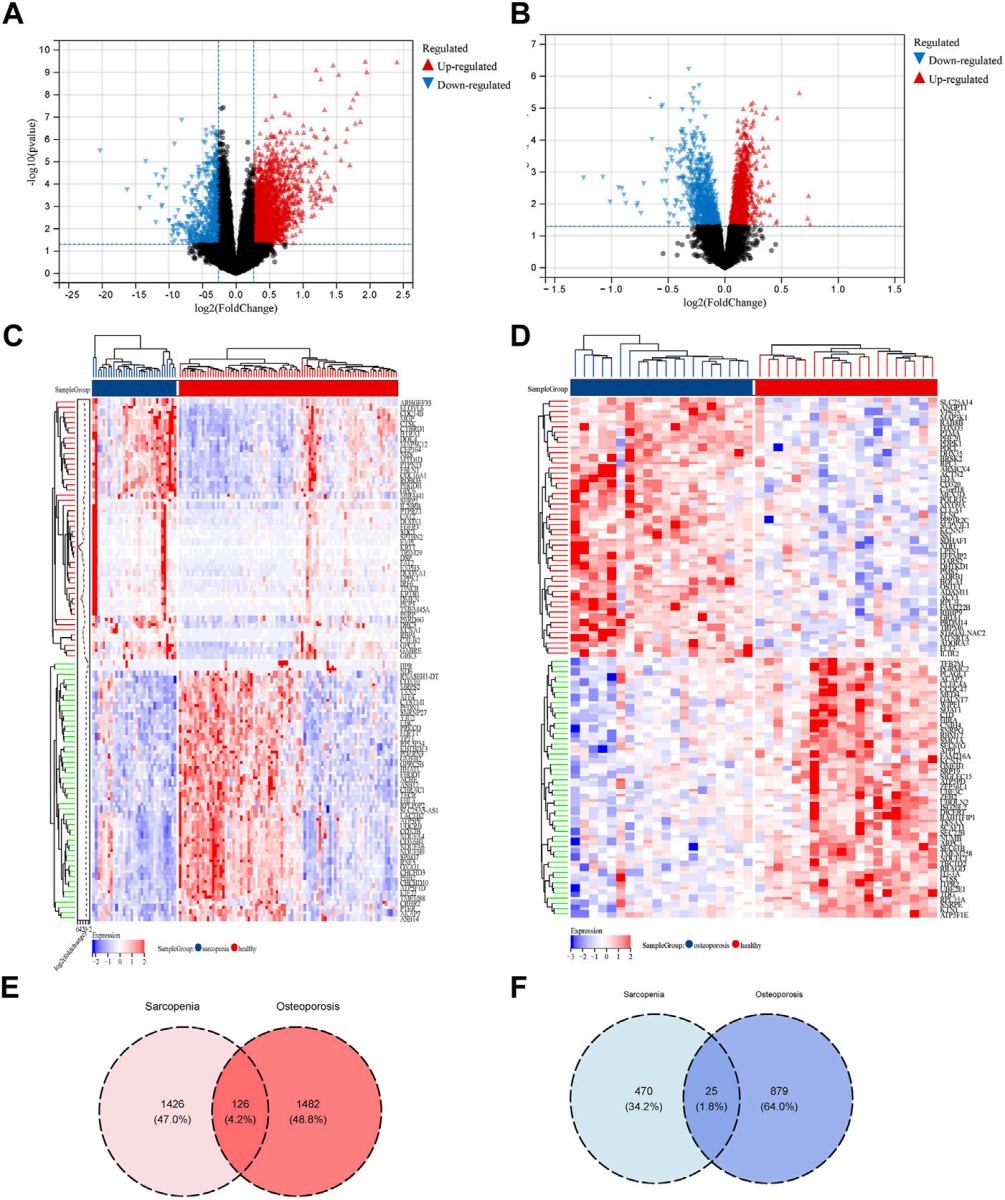
Identification of DEGs related to the co-occurrence of sarcopenia and osteoporosis. **(A)** Volcano plot of DEGs of sarcopenia: blue dots represent downregulated genes in sarcopenia, red dots represent upregulated genes. **(B)** Volcano plot of DEGs of osteoporosis: the blue dots represent downregulated genes in osteoporosis, red dots represent upregulated genes. **(C)** Heatmap of top 50 genes in sarcopenia: genes in blue were downregulated in sarcopenia, while those in red were upregulated. **(D)** Heatmap of top 50 genes in osteoporosis: genes in blue were downregulated in osteoporosis, while those in red were upregulated. **(E)** Venn diagram of the intersection of upregulated DEGs in sarcopenia and osteoporosis. **(F)** Venn diagram of the intersection of downregulated DEGs in sarcopenia and osteoporosis.

Based on the criteria that we set for p-value and fold change of DEGs, 1,426 upregulated DEGs and 470 downregulated DEGs in sarcopenia and 1,482 upregulated DEGs and 879 downregulated DEGs in osteoporosis were identified. As shown in Figures 2A–D, the expression pattern of the DEGs of sarcopenia and osteoporosis were visualized by volcano plots and heatmaps. The DEG overlap was calculated to identify the common DEGs of both diseases (Figures 2E, F). Identified for further analysis were 126 common upregulated genes and 25 common downregulated genes, as summarized in Supplementary Table S1.

### 3.3 Enrichment analysis of common DEGs

**FIGURE 3:**
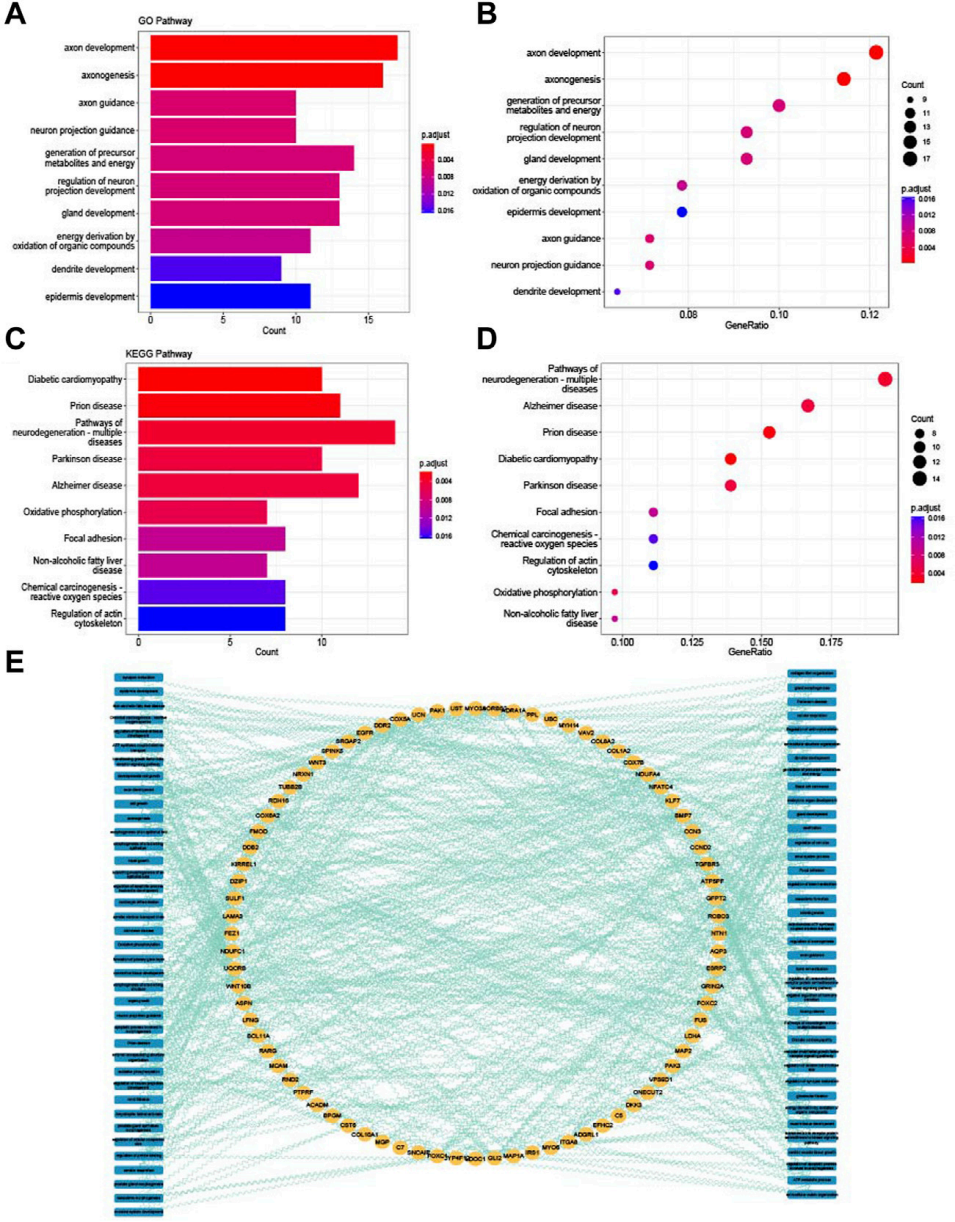
Enrichment analysis of common DEGs. **(A)** Top 10 GO BP terms of common DEGs visualized as bar plot. **(B)** Top 10 GO BP terms of common DEGs visualized as dot plot. **(C)** Top 10 KEGG terms of common DEGs visualized as bar plot. **(D)** Top 10 KEGG terms of common DEGs visualized as dot plot. **(E)** Network of relationships between common DEGs and GO and KEGG pathways.

GO enrichment and KEGG enrichment analysis were conducted to explore the biological features of common DEGs. From the enrichment analysis visualized in Figures 3A–D, many energy-related pathways were enriched in the common DEGs, such as the generation of precursor metabolites and energy derivation by oxidation of organic compounds; energy metabolism is one of the most significant pathways in the research of bone and muscle degeneration (Gomes et al., 2022; Oh et al., 2022). The network of common DEGs and both GO and KEGG terms is shown in Figure 3E; all the common DEGs and the GO and KEGG terms they enriched were connected with the genes to further understand their relationships.

### 3.4 Identification of common WGCNA genes of sarcopenia and osteoporosis

**FIGURE 4:**
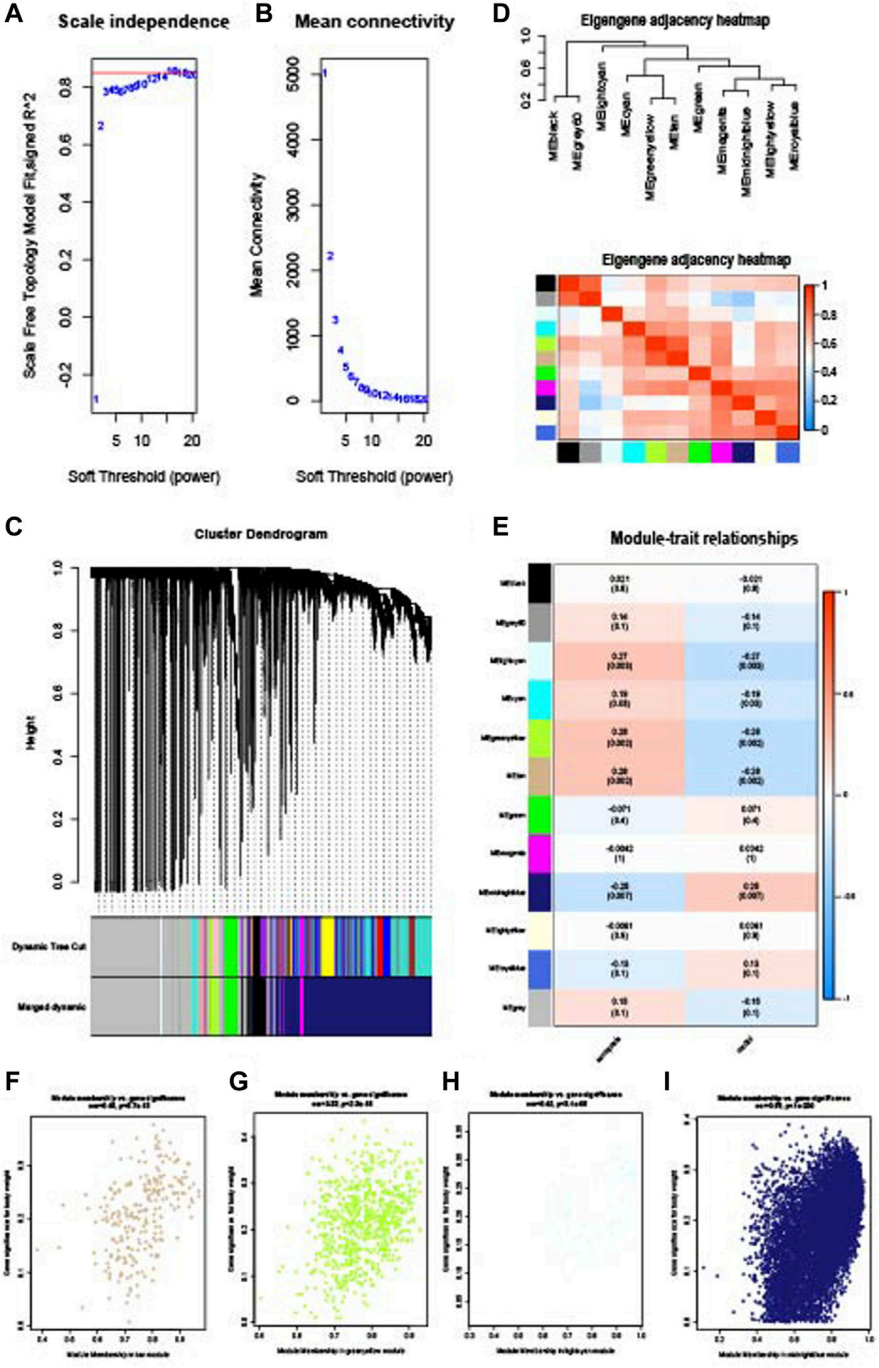
WGCNA of the sarcopenia dataset. **(A)** Selection of soft threshold. **(B)** Connectivity of different soft thresholds. **(C)** Cluster dendrogram before and after merge of modules. **(D)** Correlations of modules. **(E)** Correlations between modules and sarcopenia. **(F)** Scatter plots of gene significance in tan module. **(G)** Scatter plots of gene significance in the green–yellow module. **(H)** Scatter plots of gene significance in the light cyan module. **(I)** Scatter plots of gene significance in the midnight blue module.

**FIGURE 5:**
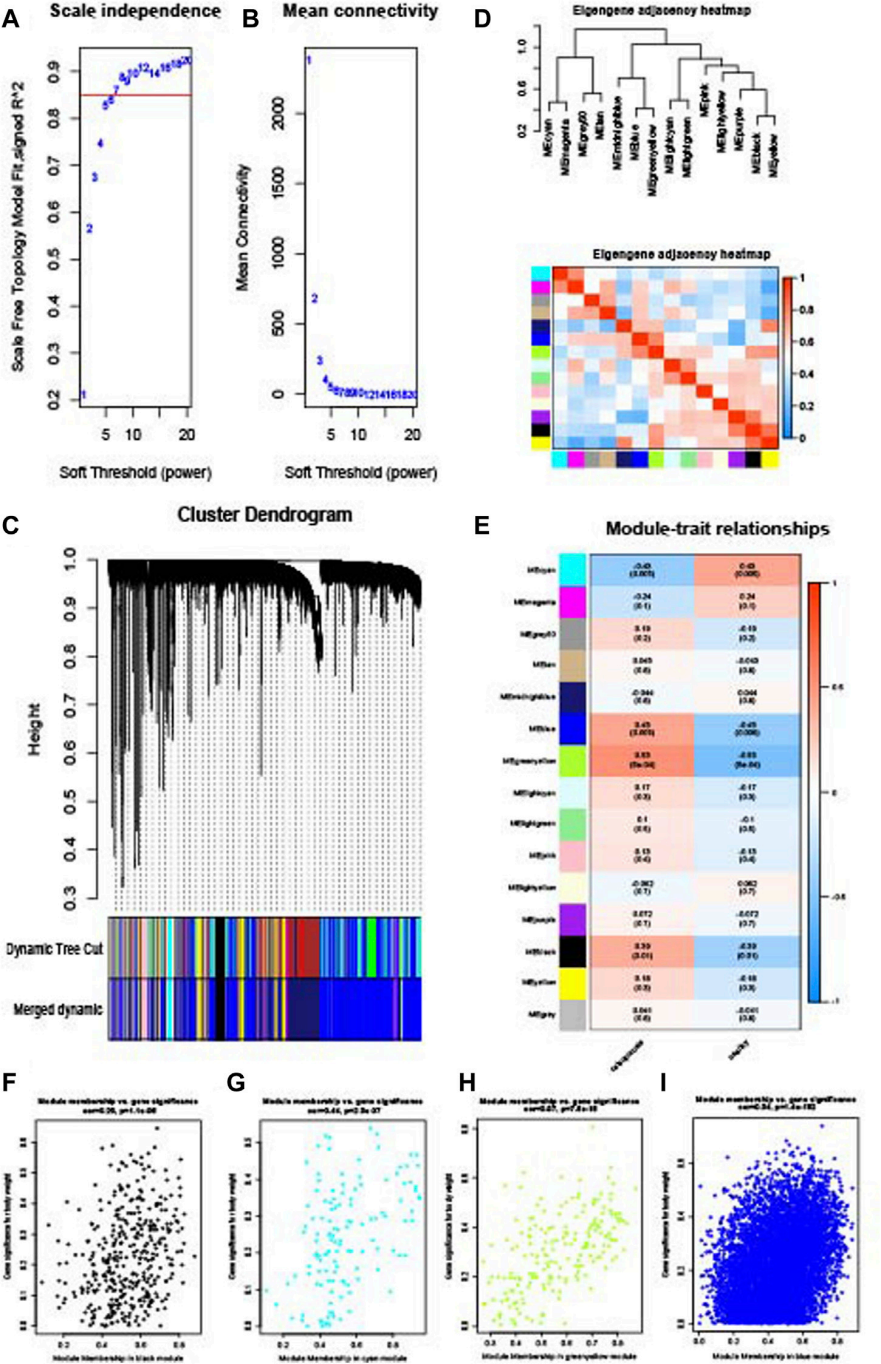
WGCNA of the osteoporosis dataset. **(A)** Selection of soft threshold. **(B)** Connectivity of different soft thresholds. **(C)** Cluster dendrogram before and after merge of modules. **(D)** Correlations of modules. **(E)** Correlations between modules and osteoporosis. **(F)** Scatter plots of gene significance in the black module. **(G)** Scatter plots of gene significance in the cyan module. **(H)** Scatter plots of gene significance in the green–yellow module. **(I)** Scatter plots of gene significance in the blue module.

**FIGURE 6:**
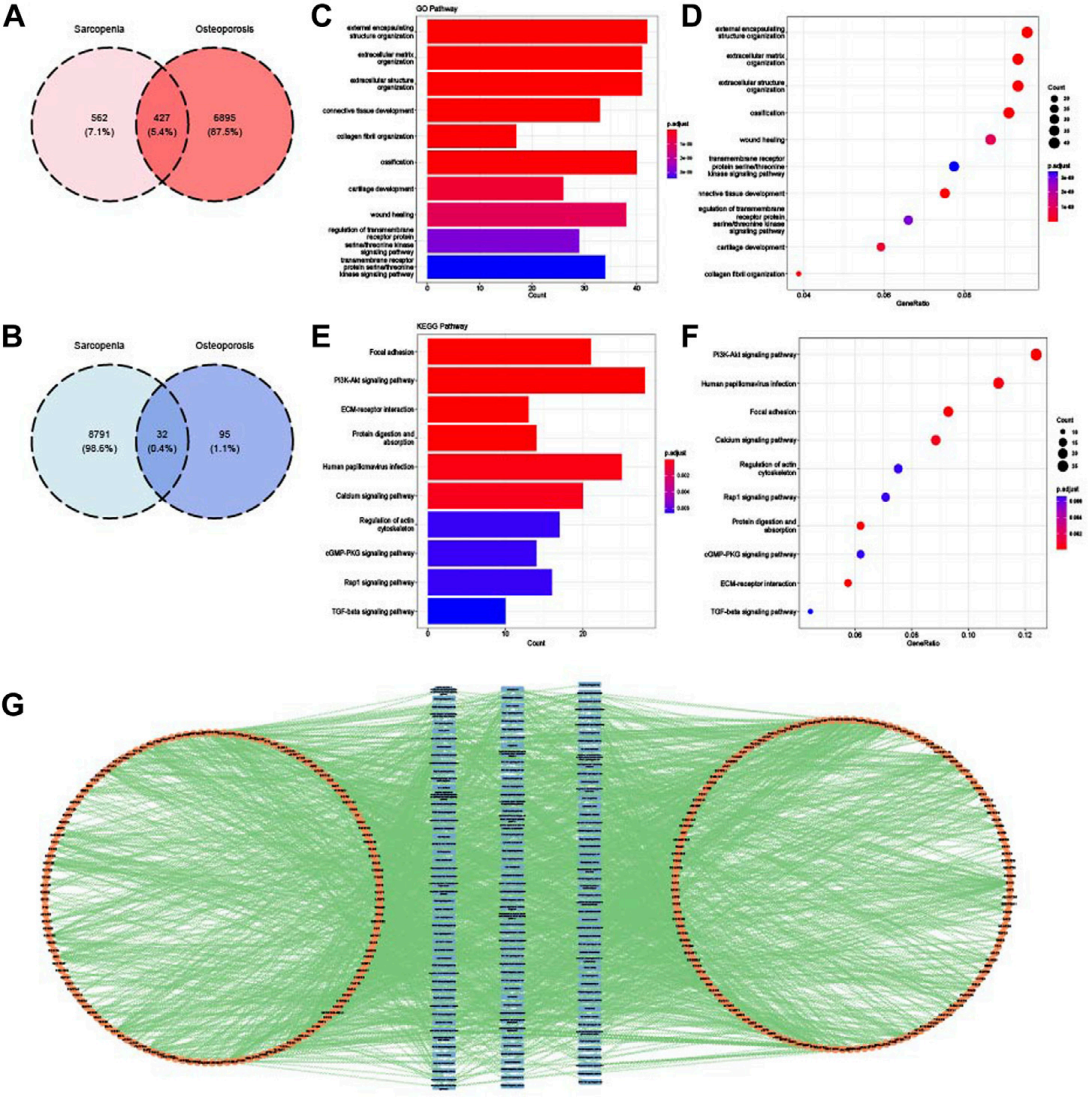
Identification and enrichment analysis of common WGCNA genes. **(A)** Venn diagram of the intersection of upregulated WGCNA module genes in sarcopenia and osteoporosis. **(B)** Venn diagram of the intersection of downregulated WGCNA module genes in sarcopenia and osteoporosis. **(C)** Top 10 GO BP terms of common WGCNA genes visualized as bar plot. **(D)** Top 10 GO BP terms of common WGCNA genes visualized as dot plot. **(E)** Top 10 KEGG terms of common WGCNA genes visualized as bar plot. **(F)** Top 10 KEGG terms of common WGCNA genes visualized as dot plot. **(G)** Network of relationships between common WGCNA genes and GO and KEGG pathways.

WGCNA analysis was performed to identify the gene modules of sarcopenia and osteoporosis. As shown in Figure 4A, the soft threshold was calculated and selected for sarcopenia; a soft threshold of 16 was identified for further analysis due to the cutoff value R^2^ = 0.90. Figure 4B shows the mean connectivity in different soft thresholds. The gene modules were then established and clustered (Figure 4C), and those with a correlation of more than 80% were merged. The relationships between different modules, as well as between modules and clinical features, are visualized in Figures 4D, E. Modules with a p-value ≤0.01 in the correlation analysis for sarcopenia were selected for further analysis (Figures 4F–I). As with the WGCNA analysis of sarcopenia, the gene modules were constructed and clustered based on the soft threshold of seven (Figures 5A–C); and correlation analysis was then performed (Figures 5D, E). The black, cyan, green, yellow, and blue modules were chosen for further analysis (Figures 5F–I). For sarcopenia, 562 up-related and 8,791 down-related module genes were identified, and 6,895 up-related and 95 down-related module genes for osteoporosis were selected. The intersection of WGCNA modules of sarcopenia and osteoporosis, containing 427 up-related genes and 32 down-related genes, was identified as common WGCNA genes (Figures 6A, B). Lists of genes in different modules of sarcopenia and osteoporosis and the common WGCNA genes are provided in Supplementary Data Sheet S1.

### 3.5 Enrichment analysis of common WGCNA genes

Biological features of common WGCNA genes were summarized and analyzed by GO and KEGG analysis. As shown in Figures 6C and D, the main pathways of common WGCNA genes were enriched in connective tissue development and extracellular function in GO analysis. Many muscle and bone metabolism signal pathways were involved for KEGG, such as the PI3K-Akt, cGMP-PKG, and TGF-beta pathways (Figures 6E, F). Moreover, consistent with the enrichment analysis of common DEGs, some energy metabolism pathways were also enriched, such as protein digestion and absorption. The relationships between pathways and genes are mapped in Figure 6G.

### 3.6 Identification and enrichment analysis of crosstalk genes

**FIGURE 7:**
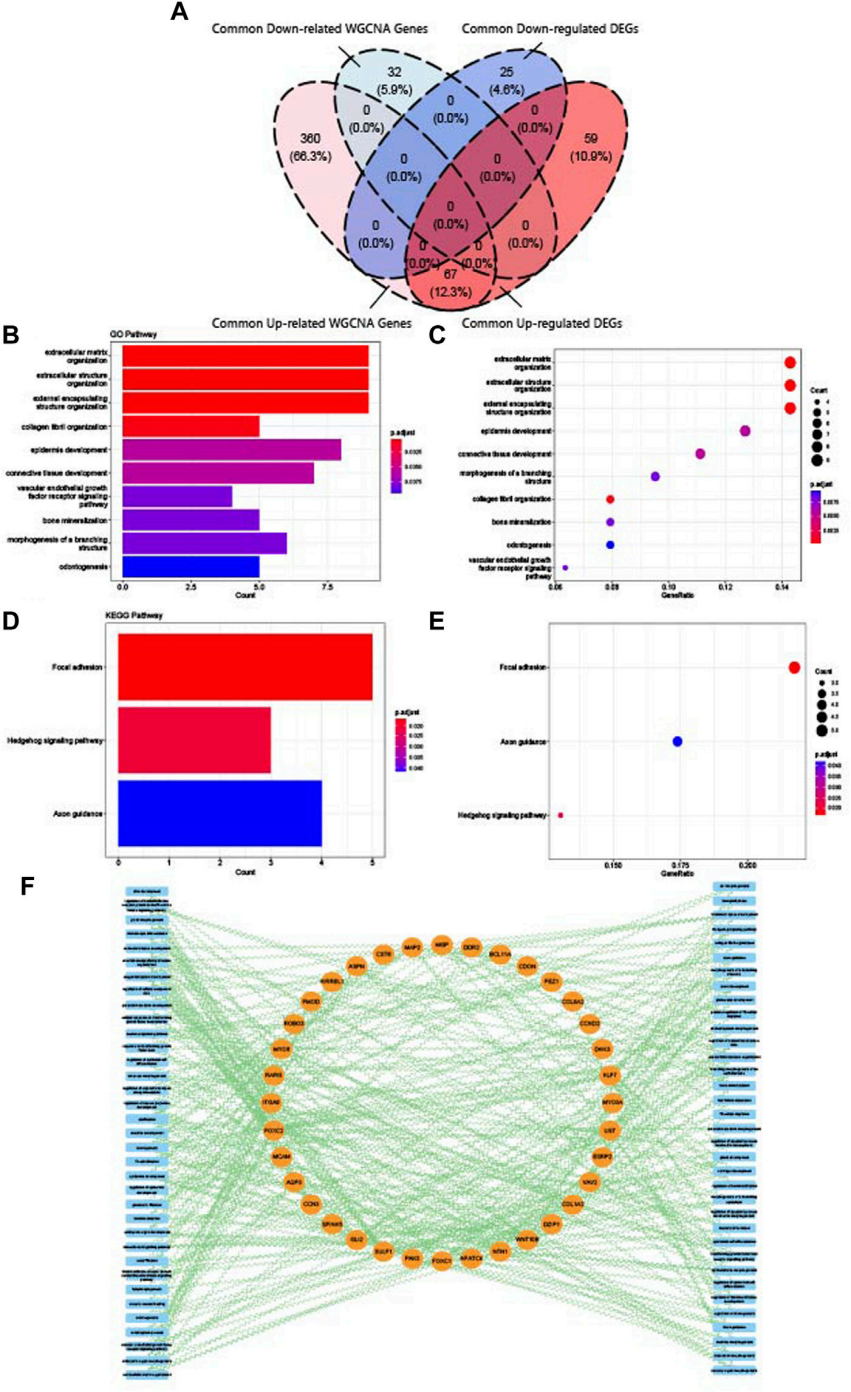
Identification and enrichment analysis of crosstalk genes. **(A)** Venn diagram of the intersection of upregulated common DEGs, downregulated common DEGs, upregulated common WGCNA genes, and downregulated common WGCNA genes. **(B)** Top 10 GO BP terms of crosstalk genes visualized as bar plot. **(C)** Top 10 GO BP terms of crosstalk genes visualized as dot plot. **(D)** Top 3 KEGG terms of crosstalk genes visualized as bar plot. **(E)** Top three KEGG terms of crosstalk genes visualized as dot plot. **(F)** Network of relationships between crosstalk genes and GO and KEGG pathways.

Finally, crosstalk genes were identified by intersecting the common DEGs and WGCNA genes. As shown in Figure 7A, 67 upregulated crosstalk genes were identified, and no downregulated genes overlapped. GO and KEGG analysis for 67 crosstalk genes were conducted and visualized in Figures 7B–E. Consistent with the results of enrichment analysis of common DEGs and common WGCNA genes, the crosstalk genes were mainly enriched in extracellular regulation, connective tissue development, vascular endothelial growth factor receptor pathway, and bone mineralization. The relationship map for enrichment analysis of crosstalk genes is shown in Figure 7F.

### 3.7 PPI network of common DEGs, common WGCNA genes, and crosstalk genes

**FIGURE 8:**
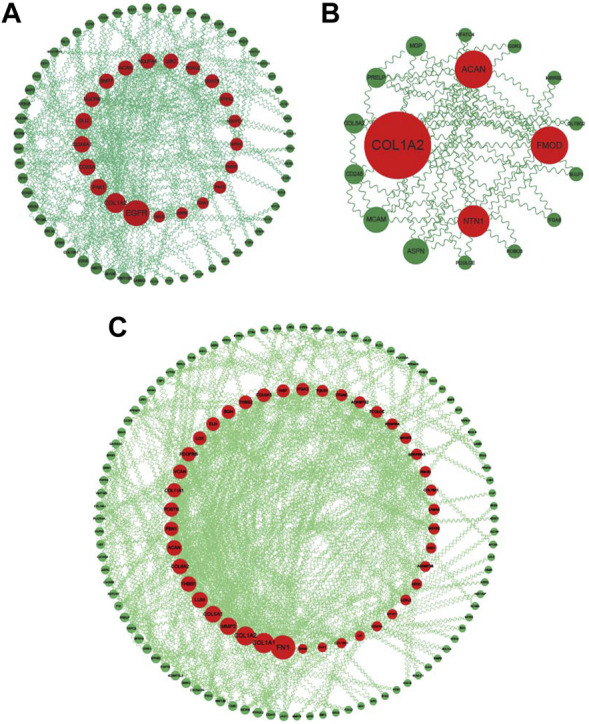
PPI networks of common DEGs, WGCNA genes, and crosstalk genes. **(A)** PPI network of common DEGs: genes ranked by size according to degree score in Cytoscape. **(B)** PPI network of common crosstalk genes: genes ranked by size according to degree score in Cytoscape. **(C)** PPI network of common WGCNA genes: genes ranked by size according to degree score in Cytoscape.

The common DEGs, common WGCNA genes, and crosstalk genes were uploaded to STRING (https://string-db.org/) to construct PPI networks and then imported to Cytoscape for visualization (Figures 8A–C). Genes were ranked according to the results of calculation by CytoNSA, with the size presenting the score calculated by the “degree” algorithm. The top five genes in the PPI network of common DEGs were epidermal growth factor receptor (EGFR), collagen type I alpha 1 chain (*COL1A1*), P21 (*RAC1*) activated kinase 1 (*PAK1*), cytochrome C oxidase subunit 5A (*COX5A*), and cytochrome C oxidase subunit 6A2 (*COX6A2*); the top five genes in PPI networks of common WGCNA were fibronectin 1 (*FN1*), *COL1A1*, collagen type I alpha 2 chain (*COL1A2*), matrix metallopeptidase 2 (*MMP2*), and collagen type V alpha 1 chain (*COL5A1*); in crosstalk, the genes were COL1A1, aggrecan (*ACAN*), fibromodulin (*FMOD*), netrin 1 (*NTN1*), and asporin (*ASPN*).

### 3.8 Identification of key crosstalk genes by machine learning

**FIGURE 9:**
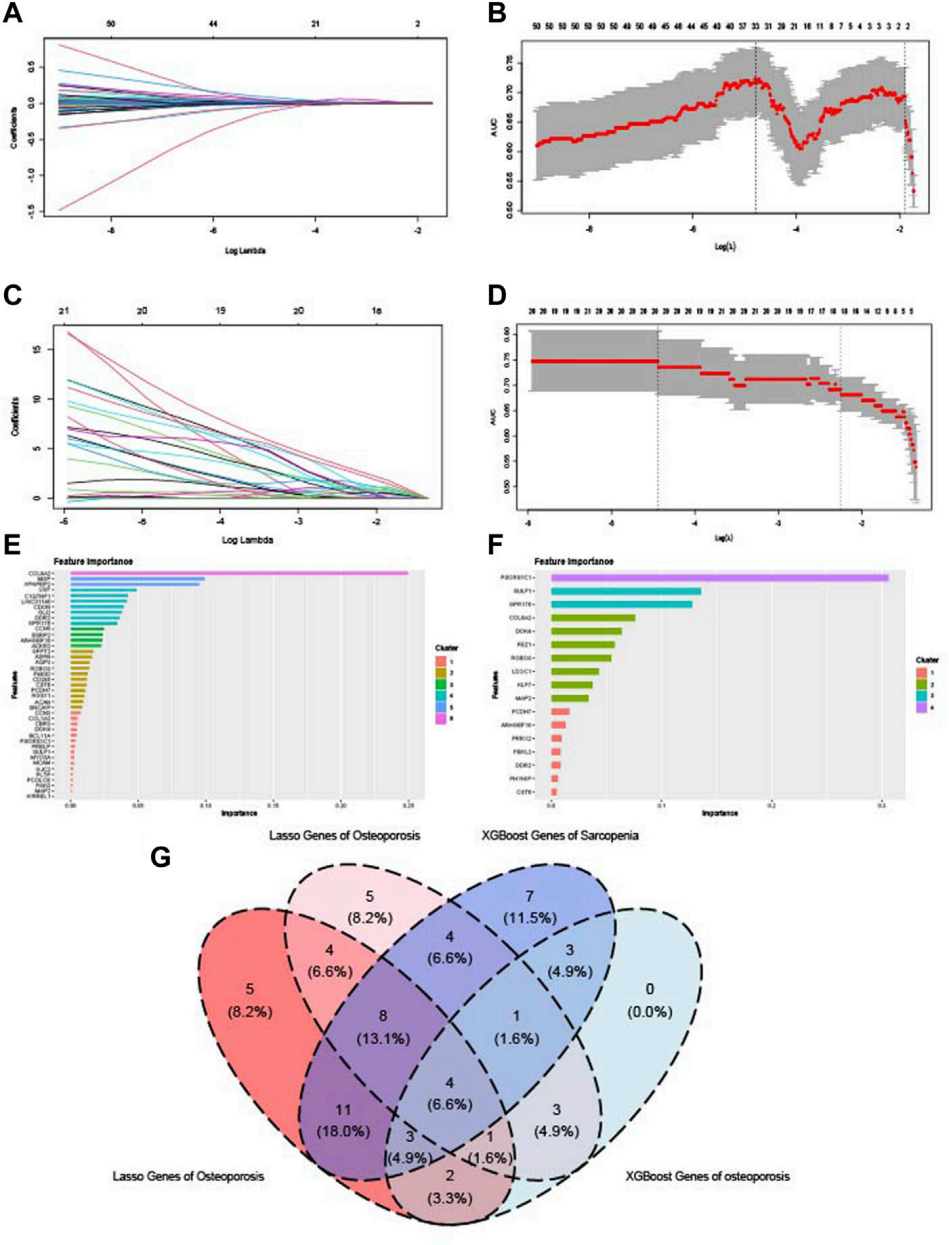
Identification of key crosstalk genes by LASSO analysis and XGBoost algorithm. **(A,B)** LASSO model constructed on the sarcopenia dataset. **(C,D)** LASSO model constructed on the osteoporosis dataset. **(E)** Importance of genes calculated by XGBoost algorithm in the sarcopenia dataset. **(F)** Importance of genes calculated by XGBoost algorithm in osteoporosis dataset. **(G)** Venn diagram of the intersection of genes with significant importance calculated by LASSO and XGBoost algorithm.

Two kinds of machine learning models were established to identify the key crosstalk genes of sarcopenia and osteoporosis. In LASSO analysis, the change trends of gene coefficients in different λ in sarcopenia and osteoporosis are shown in Figures 9A and C: as λ rises, efficiency falls to zero. The AUC of models with different λ is then calculated (Figures 9B, D), and the minimum of λ was selected to establish the models. Some 38 and 31 genes were included in the sarcopenia LASSO model and osteoporosis LASSO model, respectively (Supplementary Table S2). The XGBoost models based on the 67 crosstalk genes were constructed, and the genes’ importance in the models was calculated (Figures 9E, F). In the XGBoost models, 41 genes were included in sarcopenia models, and, in osteoporosis, 17 (Supplementary Table S3). After the intersection of the results of LASSO and XGBoost, four genes were identified as key crosstalk genes (Figure 9G): Rho guanine nucleotide exchange factor 10 (*ARHGEF10*), Protocadherin 7 (*PCDH7*), cystatin 6 (*CST6*), and roundabout guidance receptor 3 (*ROBO3*).

### 3.9 Validation of key crosstalk genes in independent datasets and clinical samples

**FIGURE 10:**
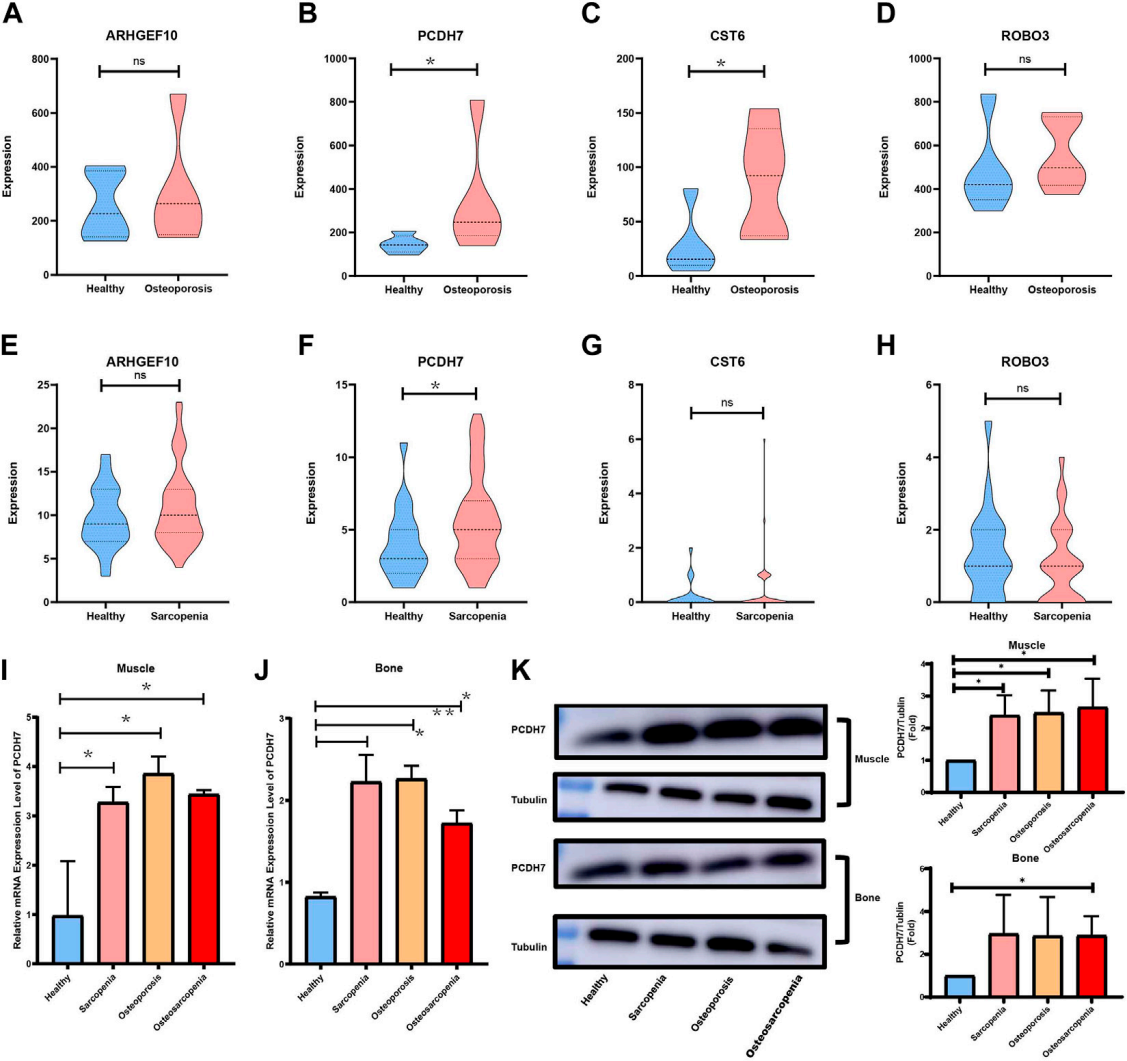
Validation of key crosstalk genes in independent datasets and experience. **(A)** Comparison of mRNA expression levels of ARHGEF10 in the sarcopenia dataset. **(B)** Comparison of mRNA expression levels of PCDH7 in the sarcopenia dataset. **(C)** Comparison of mRNA expression levels of CST6 in the sarcopenia dataset. **(D)** Comparison of mRNA expression levels of ROBO3 in the sarcopenia dataset. **(E)** Comparison of mRNA expression levels of ARHGEF10 in the osteoporosis dataset. **(F)** Comparison of mRNA expression levels of PCDH7 in the osteoporosis dataset. **(G)** Comparison of mRNA expression levels of CST6 in the osteoporosis dataset. **(H)** Comparison of mRNA expression levels of ROBO3 in the osteoporosis dataset. **(I)** Comparison of mRNA expression levels of PCDH7 in muscle samples. **(J)** Comparison of mRNA expression levels of PCDH7 in bone samples. **(K)** Protein levels of PCDH7 in muscle and bone samples.

GSE167186 and GSE35956 were used as the datasets of sarcopenia and osteoporosis to validate the key crosstalk genes. The expression level of four key crosstalk genes was compared between patients with and without osteoporosis or sarcopenia. As shown in Figures 10A–H, only PCDH7 was validated in both sarcopenia and osteoporosis test datasets. The patients were then grouped according to whether they had sarcopenia and osteoporosis, with the baseline characteristics of patients summarized in Supplementary Table S4. The mRNA expression and protein level of PCDH7 in muscle and bone tissue between patients in different groups were compared by real-time quantitative PCR and Western blot. Those patients with sarcopenia, osteoporosis, and osteosarcopenia had a significantly higher PCDH7 expression in both mRNA and protein levels than patients without sarcopenia and osteoporosis (Figures 10I–K).

### 3.10 Immune infiltration

**FIGURE 11:**
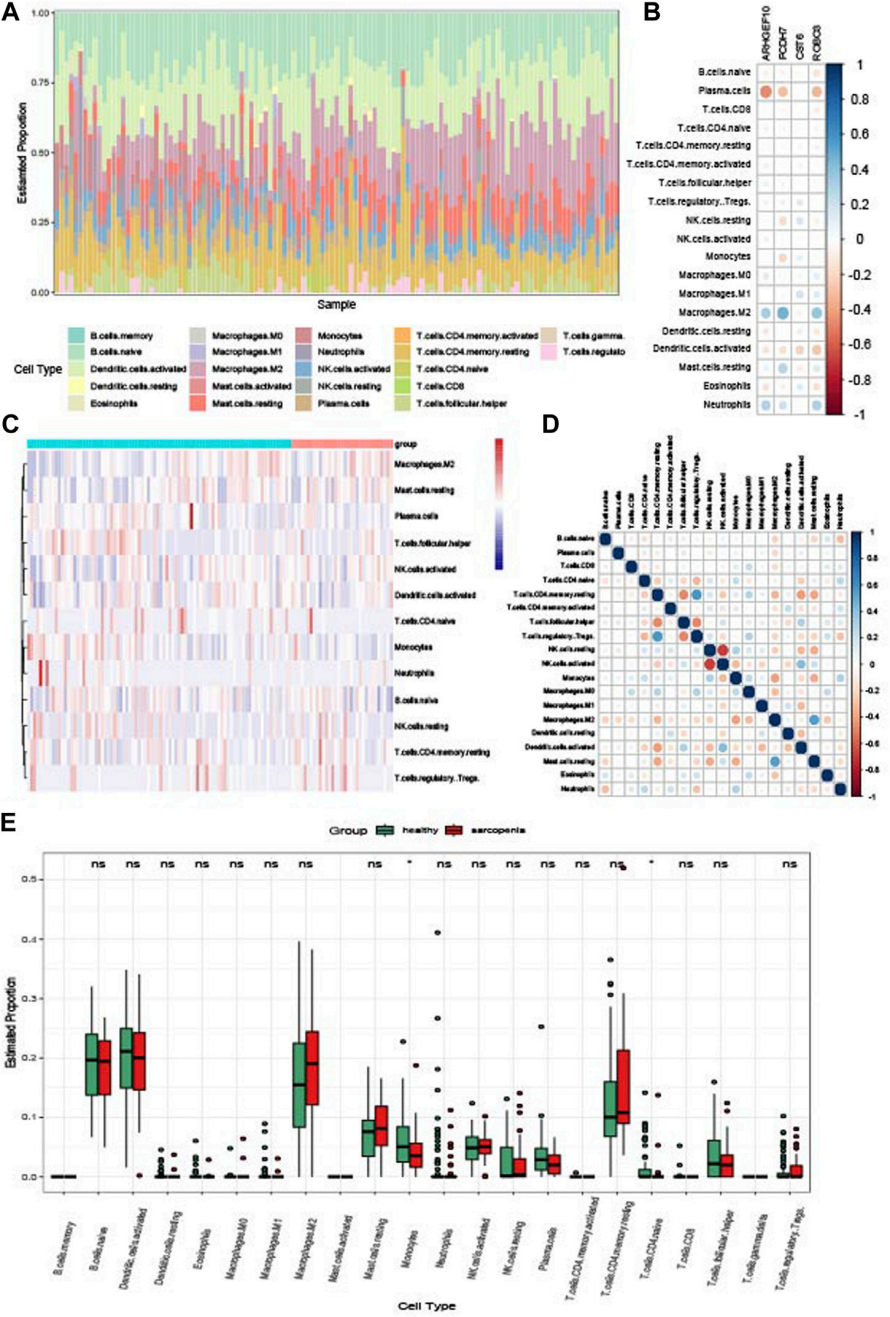
Immune infiltration analysis of sarcopenia. **(A)** Relative percentage of 22 immune cell subpopulations in the sarcopenia dataset. **(B)** Correlations between immune cell subpopulations and key crosstalk genes. **(C)** Heatmap of immune cell subpopulations in the sarcopenia dataset. **(D)** Correlations between immune cell subpopulations. **(E)** Box plot of immune cell subpopulations in the sarcopenia dataset.

To explore the relationships between the immune system and the co-occurrence of sarcopenia and osteoporosis, immune infiltration analysis was performed on the sarcopenia and osteoporosis datasets. Figure 11A shows the proportion of immune cells in different samples of the sarcopenia dataset. The relationships between key crosstalk genes and immune cells is analyzed and visualized in Figure 11B, Plasma cells and macrophages M2 were cells the most relevant to the key crosstalk genes. The relationships between sarcopenia and immune cells, as well as between immune cells, are visualized in Figures 11C and D as a heatmap and correlogram, respectively. Moreover, the levels of each type of immune cell were compared in patients with and without sarcopenia, and the levels of T cells CD4-naïve and monocytes significantly differed between groups (Figure 11E). Similarly, the results of the immune infiltration of osteoporosis are shown in Supplementary Figure S2.

## 4 Discussion

Sarcopenia and osteoporosis as degenerative diseases in the most significant part of the motor system have become severe health problems in aging societies. In this study, datasets of sarcopenia and osteoporosis were included to identify their possible crosstalk genes. The specimens of osteoporosis were blood monocytes and of sarcopenia were muscle biopsies. Monocytes as a type of circulation blood cells have been proven in many studies to relate to sarcopenia and osteoporosis (Deng et al., 2021; Nie et al., 2022; Zeng et al., 2022). Consistent with previous studies used different kind of specimens (Wang Z. et al., 2022; Liu J. et al., 2022; Mo et al., 2022), we have concluded that the intersection between gene expression maps of muscle tissues and monocytes may provide us much wider insights into the development of osteosarcopenia (Liu et al., 2017). In our study, key crosstalk genes (*ARHGEF10*, *PCDH7*, *CST6*, and *ROBO3*) were identified by DEG analysis, WGCNA, and machine learning methods, and then validated by independent datasets and clinical samples. Finally, *PCDH7* was identified and validated as the gene that may be significantly related to the co-occurrence of sarcopenia and osteoporosis.

The pathogenesis behind sarcopenia is still a controversial topic and is hotly debated. It is widely believed that the skeletal muscle mass in humans peaks at age 30 and then decreases significantly with age, while, in individuals aged over 75, skeletal muscle accounts for only a quarter of total body weight (Weitao Shen et al., 2022). Moreover, myodynamia may face a more severe decrease than muscle mass, and it is estimated that muscle strength declines at about two to five times the rate of muscle mass loss (Tournadre et al., 2019). Many studies have demonstrated that the skeletal muscle satellite cells—myogenic stem cells in skeletal muscles—play a significant role in the regeneration and recovery of muscle by regulating their proliferation and differentiation, and that the degeneration in both quantity and quality of satellite cells may relate to the development of sarcopenia (Hirano et al., 2023). Satellite cells from young mice may have a more sensitive and quicker ability to activate their cell cycle of proliferation than those from older mice when subject to external stimuli on a muscle (Leiter et al., 2011). However, the disability of satellite cells may be the result of sarcopenia but not a contributor to its development. Researchers have tried to transplant the muscle samples from cadavers of different ages to immunodeficient mice, and, interestingly, there was no significant difference in myogenic capacity (Novak et al., 2021). Therefore, the development of sarcopenia might not be caused by the degeneration of satellite cells but might be attributable to progressive and detrimental changes in the muscle microenvironment.

Many factors may influence the homeostasis of the muscle microenvironment. Neuromuscular junction as highly specialized chemical synapses formed between motor neurons and skeletal muscle fibers plays an important role in maintaining muscle tonus and preventing muscular atrophy (Cheng et al., 2022). The function of the neuromuscular junction was mainly achieved by regulating the release of acetylcholine (ACh) and the activity of ACh receptors (AChRs); in older adults, agrin, an important factor in Agrin-Lrp4-MuSK signaling pathway regulating AChR, may face a structural abnormality and then contribute to the scrambled AChR localization and dysfunctional neuromuscular junction (Butikofer et al., 2011; Gros et al., 2022). Moreover, α-synuclein, a protein localized in motor neuron axons and neuromuscular junction, may also increase myofiber apoptosis by inhibiting the release of Ach and aggregating oxidative stress (Yang et al., 2022). Many researchers believe that mitochondrial dysfunction may be another factor in the development of sarcopenia. Within aging bodies, severe mitochondrial oxidative injury may cause dysfunction of the calcium signaling pathway and then induce sarcopenia (Cheema et al., 2015). Many downstream pathways and factors relating to muscle regeneration may be regulated and activated by mitochondria, including the mammalian target of rapamycin complex 1 (mTORC1), Kelch-like ECH-associated protein 1 (KEAP1), and NF-E2-related factor 2 (NRF2) (Rai and Dey, 2020). The mammalian target of rapamycin (mTOR) has been proven to be the key regulator of muscle regeneration and to regulate bone metabolism by stimulating protein synthesis and inhibiting proteolysis (Sirago et al., 2022). Inflammaging, a chronic inflammatory phenomenon occurring in older individuals and characterized by an increase in proinflammatory cytokines and a decrease in anti-inflammatory cytokine levels, was also a cause of many geriatric syndromes, including sarcopenia (Bencivenga et al., 2022). With inflammation, the excessive release of inflammatory factors, such as tumor necrosis factor-alpha (TNF-α), interleukin-6 (IL-6), and C-reaction protein (CRP), may influence the capacity of satellite cells and contribute to the disorder of the muscle microenvironment (Savary-Auzeloux et al., 2022). TNF-α may induce the overexpression of muscle atrophy-related genes, such as *MuRF-1* and *MAFbx*, by regulating the TNF receptor-associated factor 6 (TRAF6) and activating the nuclear factor-kappa B (NF-κB) signaling pathway (Li et al., 2020). CRP may negatively affect muscle cell size directly by reducing the phosphorylation of protein kinase B (AKT) and ribosome protein subunit 6 kinase 1 (S6K1) (Oflazoglu et al., 2022).

Osteoporosis, one of the most common geriatric syndromes of older adults and postmenopausal women, has also attracted increasing attention from researchers around the world. It was widely believed that the imbalance between osteoclasts and osteoblasts contributed significantly to the development of osteoporosis, and may affect bone metabolism by regulating the activity of osteoclasts and osteoblasts (Yan et al., 2022). Many signaling pathways involved in osteoporosis have been explored, including OPG/RANKL/RANK, Wnt/β-catenin, PI3K/Akt/mTOR, MAPK, and Hedgehog (Patel and Wairkar, 2022). OPG/RANKL/RANK is one of the most significant signaling pathways in the development of osteoporosis: osteoprotegerin (OPG), a factor secreted by osteoblasts, and a receptor activator of NF-κB ligand (RANKL) competitively bind to the receptor activator of NF-κB (RANK) (Wang F. et al., 2022). Many factors may affect bone metabolism by regulating OPG/RANKL/RANK, including parathyroid hormone (PTH), vitamin D, prostaglandin, and estrogen (Chen et al., 2018). Wnt/β-catenin is a key pathway involved in the osteogenic differentiation of mesenchymal stem cells (Marini et al., 2022). It has been found that the Wnt/β⁃catenin signal directly induces BMP-2 expression and enhanced BMP-2 transcription activity in osteoblasts via the TCF/LEF reaction element, and that β-catenin can enhance the sensitivity of mesenchymal stem cells to BMP-2, inducing its differentiation into osteoblasts and promoting its formation (Huybrechts et al., 2020; Xie et al., 2021). The PI3K/Akt signaling pathway is mediated by enzyme-linked receptors (Yang et al., 2022). PI3K/Akt can activate the signaling pathway with the participation of various growth factors, cytokines, and extracellular matrix and also plays an important role in cell proliferation, apoptosis, tissue inflammation, and tumor growth and invasion (Wang Q. et al., 2022; Hashemi et al., 2022; Quan et al., 2022). Insulin-like growth factor-1(IGF-1) as well as vascular endothelial growth factor (VEGF) may promote the differentiation of bone marrow mesenchymal stem cells into osteoblasts by regulating the PI3K/Akt/mTOR signaling pathway (Karar and Maity, 2011; Bakker et al., 2016).

The interaction between bone metabolism and the immune system was also an interesting topic of osteoporosis (Terashima and Takayanagi, 2018); we therefore conducted immune infiltration to explore their relationships. In previous studies, OPG/RANKL/RANK had been reported to have an important role in osteoimmunology due to its impact on the differentiation from monocytes to osteoclasts (Okamoto and Takayanagi, 2019). Many cells involved in the immune function express RANK and RANKL, and many factors may play a role in both immune activities and bone metabolism. M1/M2 dichotomy is a commonly known immune cell due to its impact on the bone microenvironment, and, with a decreased of estrogen level and increased RANKL level, the M1/M2 ratio was influenced (Yang et al., 2019). Similarly, immune cytokines including TNF-α, IFN-γ, IL-1, IL-4, IL-6, and IL-17, may also affect the skeletal system (Zhou et al., 2021).

Both bone and muscle are derived from the mesenchymal stem cells of the mesoderm and ectoderm; the anatomical relationships between them provide more possible interactions of mechanical and chemical signals (Brotto and Bonewald, 2015). Decreased muscle mass may promote the loss of bone mass, while the muscle contractions may partially inhibit bone loss and trabecular structural degradation due to a lack of daily weight-bearing activities (Lam and Qin, 2008; Manske et al., 2011). Myostatin, a member of the TGF-β superfamily secreted by skeletal muscle cells, also inhibits the increase of muscle and bone mass (Buehring and Binkley, 2013). Similarly, Indian hedgehog (Ihh), a factor secreted by osteoblast, may regulate muscle function and mass (Bren-Mattison et al., 2011). Moreover, the gap junction protein connexin 43 also affects both bone and muscle: the connexin 43 gene ablation mouse may have lower muscle weight and strength and defective skeletal development (Shen et al., 2015).

Extracellular matrix organization and structure, connective tissue development, VEGF signaling pathway, focal adhesion, and hedgehog signaling pathway, as we showed in the enrichment analysis of crosstalk genes, are all pathways involved in the regulation of the connective tissue microenvironment. Consistent with the mechanism of bone and muscle mentioned previously, the signaling pathway between bone and muscle plays an important role in the development of musculoskeletal disorders. Therefore, there must be some genes that regulate both bone and muscle development, as well as the interaction between them, and contribute to the co-occurrence of sarcopenia and osteoporosis.

By using the bioinformatic methods, this study identified PCDH7 as the crosstalk gene that bridges sarcopenia and osteoporosis—validated by real-time quantitative PCR and Western blot on clinical samples. Cadherin and its related proteins constitute a cadherin superfamily, a group of calcium-dependent transmembrane glycoproteins that mediate cell–cell adhesion and bonding in tissues (Hatta and Takeichi, 1986). The cadherin superfamily is roughly divided into four categories: classical cadherins, pontoon cadherins, protocadherins (PCDHs), and other types of cadherin-related proteins (Shirayoshi et al., 1986). PCDH7 is a member of the PCDH family, the largest subfamily of cadherin, and belongs to the PCDHδ1 family (Yoshida et al., 1999). The extracellular domain of PCDH7 consists of seven repeats of the cadherin motif (EC1–7), and the EC2 of PCDH7 is unique due to its 55-amino-acid insertion into the middle of the motif (Yoshida et al., 1998). PCDH7 plays a role in many cancers, including lung adenocarcinoma (Ma et al., 2022) and gastric cancer (Xin et al., 2022), colon (Liu Z. et al., 2022), ovarian (Salgado-Albarran et al., 2022), and breast cancers (Zhang X. et al., 2022). Moreover, many studies have reported its impact on physiological processes, such as dendritic spine morphology, synaptic function, and the formation of homotypic cell-in-cell structures (Wang C. et al., 2020; Wang Y. et al., 2020).

The relationships between PCDH7 and sarcopenia, as well as osteoporosis, are not fully understood. Several studies have reported that PCDH7 may affect bone metabolism by regulating osteoclast differentiation: the bone marrow-derived monocytes retrovirally transduced with shRNA for PCDH7 may face significantly reduced osteoclast maturation; similarly, PCDH7-deficient mice had impaired osteoclast differentiation but increased bone mass and normal osteoblast function (Kim et al., 2020). However, few studies have reported whether PCDH7 plays a role in muscle development and atrophy, as well as whether PCDH7 contributes to the development of osteoporosis.

Three other key crosstalk genes were also identified: *ARHGEF10*, *CST6*, and *ROBO3*, which may contribute to bone and muscle metabolism. ARHGEF10 regulates the function of vascular endothelial cells by contributing to cell junction disruption and reorientation (Abiko et al., 2015; Khan et al., 2021), and vascular function plays significant role in bone regeneration after damage (Gautvik et al., 2022; Qin et al., 2022). CST6, an extracellular polypeptide inhibitor of cysteine proteases, also affects bone metastasis by regulating osteoclastogenesis in many cancers, such as breast cancer and multiple myeloma (Li et al., 2021; Gai et al., 2022). ROBO3 is a member of the ROBO protein family, a subfamily of the immunoglobulin transmembrane receptor superfamily (Pak et al., 2020). ROBO proteins affect bone metabolism through Slit-ROBO signaling and neural epidermal growth factor-like proteins (Niimi, 2021), as well as muscle patterning and vascular injury regulation (Long and Van Vactor, 2012; Yuen and Robinson, 2013; Ordan and Volk, 2015).

After validation by independent datasets, only PCDH7 was selected for further validation due to its significantly different expression level in test datasets. To prove the relationship between PCDH7 and osteosarcopenia, we collected clinical samples of patients with sarcopenia, osteoporosis, both sarcopenia and osteoporosis, and patients without sarcopenia or osteoporosis. Gene expression and protein levels were detected and compared; consistent with the results, patients with sarcopenia, osteoporosis, or both have a significantly higher level of PCDH7 than healthy adults. This suggests that PCDH7 must play a role in the development of both sarcopenia and osteoporosis—but how? More research is needed to explore the mechanism behind them and to provide stronger evidence.

## 5 Conclusion

Of four key crosstalk genes (*ARHGEF10*, *PCDH7*, *CST6*, and *ROBO3*), PCDH7 was identified and validated as relating the development of both sarcopenia and osteoporosis. This may provide new insights for treating older patients with sarcopenia and osteoporosis.

## Data Availability

The original contributions presented in the study are included in the article/Supplementary Material; further inquiries can be directed to the corresponding authors.
