# Supplementary figures and images for: PCDH7 as the key gene related to the co-occurrence of sarcopenia and osteoporosis
Source: Front Genet. 2023 Jul 5;14:1163162. doi: 10.3389/fgene.2023.1163162 (PMC10354703; doi:10.3389/fgene.2023.1163162)

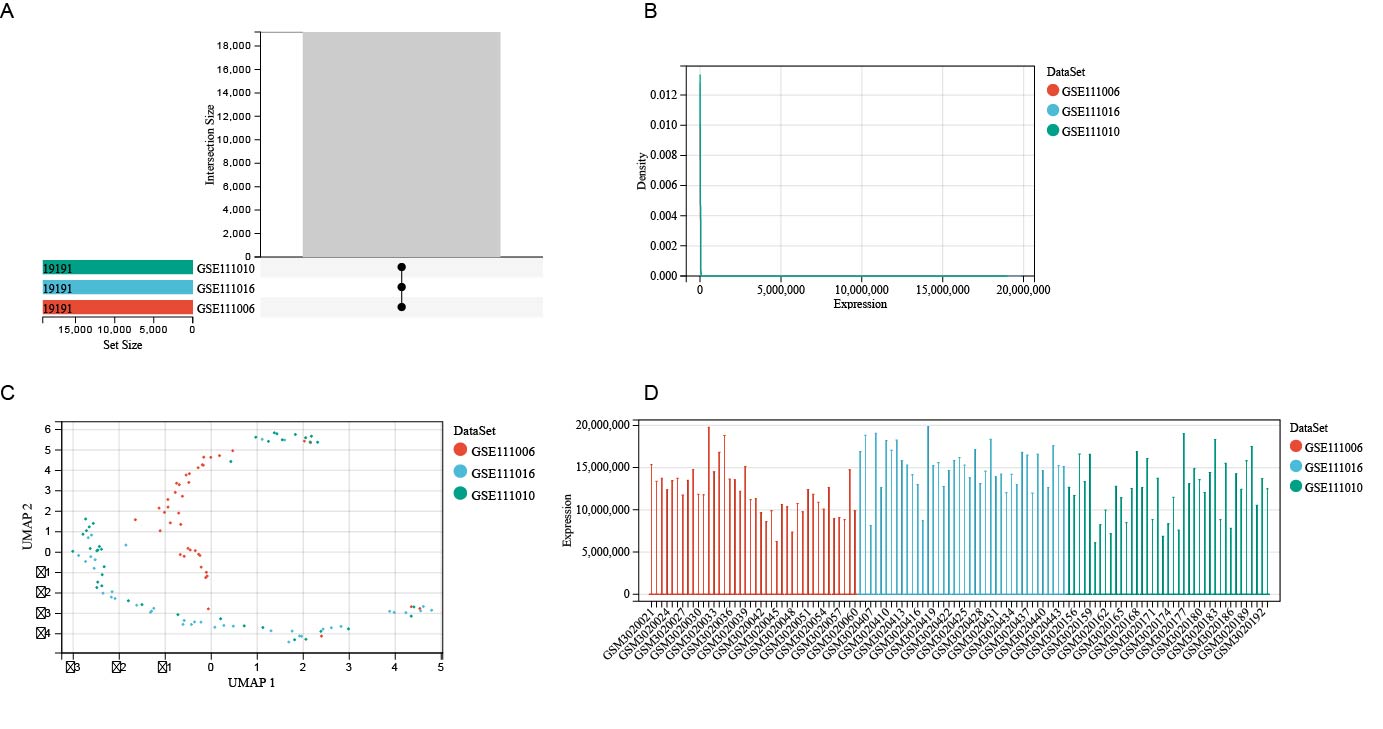

Supplement: Supplementary file 3 [file Image1.JPEG]

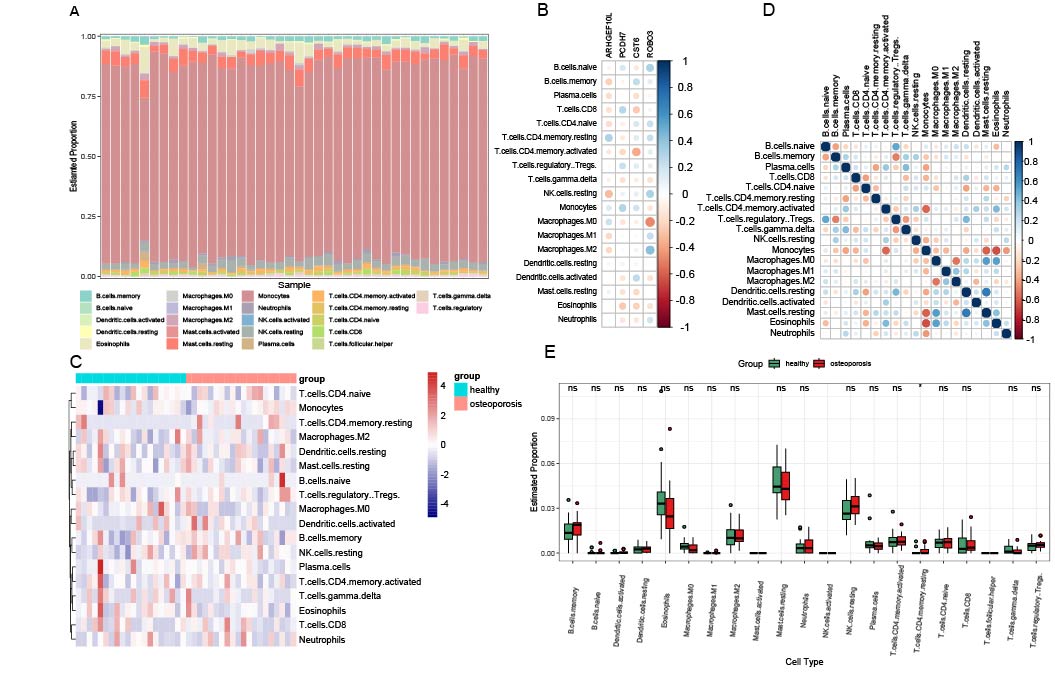

Supplement: Supplementary file 4 [file Image2.JPEG]
